# Where to Start the Journey to Advance Age Inclusivity at Your Institution

**DOI:** 10.1093/geroni/igab046.864

**Published:** 2021-12-17

**Authors:** Michelle Porter, Elizabeth Bergman

**Affiliations:** 1 University of Manitoba, Centre on Aging, Winnipeg, Manitoba, Canada; 2 Ithaca College Gerontology Institute, Ithaca, New York, United States

## Abstract

Each institution’s journey to becoming more age inclusive will to depend on its unique characteristics, and be dependent on its strengths and existing gaps. A good place to start is to explore how to build connections and leverage existing initiatives, such as research programs, community connections and importantly the institution’s strategic plan. At this point, elements to consider include coalition building, identifying strengths and gaps, and reframing aging. Because ageism can be a hindrance in many ways, strategies to address ageism should be included. GSA initiatives and tools such as the Reframing Aging Initiative, Ageism First Aid and AARPs Disrupt Aging will be highlighted in our presentation. Examples of how several universities have charted their course to becoming more age-inclusive and age-friendly will be outlined.

